# Downregulation of Long Non-coding RNA *FALEC* Inhibits Gastric Cancer Cell Migration and Invasion Through Impairing *ECM1* Expression by Exerting Its Enhancer-Like Function

**DOI:** 10.3389/fgene.2019.00255

**Published:** 2019-03-22

**Authors:** Huazhang Wu, Fengchang Qiao, Yunli Zhao, Shouwei Wu, Minjie Hu, Tao Wu, Fuxin Huang, Wenjing Chen, Dengzhong Sun, Mulin Liu, Jinsong Zhao

**Affiliations:** ^1^School of Life Sciences, Anhui Province Key Laboratory of Translational Cancer Research, Bengbu Medical College, Bengbu, China; ^2^Department of Prenatal Diagnosis, The Affiliated Obstetrics and Gynecology Hospital of Nanjing Medical University, Nanjing Maternity and Child Health Care Hospital, Nanjing, China; ^3^School of Public Health, Bengbu Medical College, Bengbu, China; ^4^Department of Gastrointestinal Surgery, The First Affiliated Hospital of Bengbu Medical College, Bengbu, China; ^5^Department of Basic Medicine, Biochemistry Teaching and Research Section, Wannan Medical College, Wuhu, China

**Keywords:** lncRNA, *FALEC*, *ECM1*, migration and invasion, gastric cancer

## Abstract

Long non-coding RNAs (lncRNAs) have been shown to play important roles in many human diseases. However, their functions and mechanisms in tumorigenesis and development remain largely unknown. Here, we demonstrated that focally amplified lncRNA in epithelial cancer (FALEC) was upregulated and significantly correlated with lymph node metastasis, TNM stage in gastric cancer (GC). Further experiments revealed that FALEC knockdown significantly inhibited GC cells migration and invasion *in vitro.* Mechanistic investigations demonstrated that small interfering RNA-induced silencing of FALEC decreased expression of the nearby gene extracellular matrix protein 1 (*ECM1*) in *cis.* Additionally, *ECM1* and *FALEC* expression were positively correlated, and high levels of ECM1 predicted shorter survival time in GC patients. Our results suggest that the downregulation of *FALEC* significantly inhibited the migration and invasion of GC cells through impairing *ECM1* expression by exerting an enhancer-like function. Our work provides valuable information and a novel promising target for developing new therapeutic strategies in GC.

## Introduction

Gastric cancer (GC) is the third leading cause of cancer-related death worldwide, and imposes a significant health burden worldwide, especially in East Asia (Rahman et al., 2014; Ferlay et al., 2015; Torre et al., 2015). In spite of the rapid advances in medical technology such as surgery, radiation and chemotherapy during recent decades, GC patients are usually diagnosed at an advanced stage with distant metastases which results in a low 5-year survival rate (Macdonald et al., 2001; Gupta and Massague, 2006; Ferlay et al., 2015). Metastasis is responsible for as much as 90% of cancer-associated mortality, yet it remains one of the most enigmatic aspects and is a poorly understood component of cancer pathogenesis (Chaffer and Weinberg, 2011). Therefore, identifying metastasis-related genes and unraveling their underlying molecular mechanism involved in metastasis will provide potential molecular targets and may help deliver new effective therapies for GC patients with pre-established metastases.

Carcinogenesis and development of GC are complex biological process characterized by various molecular abnormalities, including both genetic and epigenetic alterations. With the advancement of high-throughput sequencing technology, common long non-coding RNA species exceeding 200 nucleotides in length and without protein-coding potential have recently aroused great interest (Ponting et al., 2009). Mounting evidences have indicated that lncRNAs involved in the occurrence and development of malignant tumors (Guttman et al., 2011; Wang and Chang, 2011; Schmitt and Chang, 2016) through a variety of mechanisms, including chromosome remodeling, RNA processing and stability, transcription and post-transcriptional regulation (Kapranov et al., 2007; Khalil et al., 2009; Cesana et al., 2011; Heo and Sung, 2011; Lee, 2012; Xing et al., 2014). Indeed, several lncRNAs associated with tumor development and progression such as *HOTAIR, ANRIL, H19, PVT1* and *GAPLINC*, have been well characterized in GC and shown to have aberrant expression (Hu Y. et al., 2014; Li et al., 2014; Fang et al., 2015; Nasrollahzadeh-Khakiani et al., 2017). However, although the functions of lncRNAs are gradually being understood, the underlying mechanisms of lncRNA-related regulation are still remains largely uncharacterized.

Recently, non-coding RNAs with enhancer-like (eRNA) activity have become one of the most interesting fields in the understanding of gene transcription because of their wide transcription from enhancer DNA elements (Kim et al., 2010; Lam et al., 2014; Mao et al., 2018), and the knockdown of eRNAs would decrease nearby or target gene expression by *cis*-regulation (Orom et al., 2010). The enhancer-like lncRNA *FALEC*, located in close proximity to the tumor metastasis-associated *ECM1* gene, has attracted our attention (Orom et al., 2010; Lee et al., 2015). Retrospective analysis showed that *FALEC* contributed to carcinogenesis by regulating the expression of *AKT1, p21, E2F*, and acting as a ceRNA of *miR-1236*, affecting the *PTEN/AKT* signaling pathway and targeting epithelial-mesenchymal transition-related genes by *trans*-regulation (Hu X. et al., 2014; Jeong et al., 2016; Pan et al., 2017; Li B. et al., 2018; Li Y. et al., 2018; Wang et al., 2018). In the present study, we found that the expression of *FALEC* was significantly upregulated in GC tissues compared with paired adjacent non-tumor tissues. RNA interference-based loss-of-function assays found that *FALEC* knockdown inhibited the migration and invasion ability of GC cells through impairing *ECM1* expression by exerting its enhancer-like function in *cis*. Our results not only reveal a novel molecular regulatory mechanism of *FALEC* in GC, but also indicate a potential biomarker and therapeutic target for individualized treatment of GC patients.

## Materials and Methods

### Tissue Collection and Ethics Statement

A total of 60 matched pairs of GC tissues and adjacent normal tissues were obtained from patients who underwent surgical resection without any neoadjuvant treatment between May 2016 and July 2018 at the First Affiliated Hospital of Wannan Medical College, China. Tumor–node–metastasis (TNM) staging of GC samples was performed by two senior pathologists. All tissue specimens after surgery were immediately frozen in liquid nitrogen for subsequent RNA isolation. Written informed consent was obtained from all GC patients, and the study was performed with the approval of the Medical Ethical Committee of Wannan Medical College.

### Cells Culture and Transfection

The human GC cell lines SGC-7901, BGC-823, AGS, MKN-45, MGC-803, HGC-27 and the immortalized human gastric epithelial mucosa cell line GES-1 were purchased from the Institute of Biochemistry and Cell Biology of the Chinese Academy of Sciences (Shanghai, China) and the Cell Resource Center of Institute of Basic Medical Sciences, Chinese Academy of Medical Sciences and Peking Union Medical College (Beijing, China). Cells were maintained in RPMI 1640 medium supplemented with 10% fetal bovine serum (FBS, Wisent, St-Bruno, PQ, Canada), 100 U/ml penicillin and 100 mg/ml streptomycin (Invitrogen, Carlsbad, CA, United States) in a 37°C incubator supplied with humidified air containing 5% CO_2_. SiRNA and antisense oligodeoxy nucleotides (ASO) sequences were designed and synthesized by GenePharma (GenePharma, Shanghai, China) and RiboBio (RiboBio, Ltd., Guangzhou, China), all the sequences for siRNAs and ASO were listed in Supplementary Table S1. GC cells were transfected with the siRNA at a final concentration of 25 nmol/L using Lipofectamine 2000 (Invitrogen, CA, United States) and ASOs at the final concentration of 100 nM using Oligofectamine transfection reagent (Invitrogen) according to the manufacturer’s instructions.

### RNA Extraction, cDNA Synthesis and Quantitative Real-Time (qRT) PCR Analysis

DNA extraction of GC cells was performed with the Genomic DNA Mini Preparation Kit (Beyotime Biotechnology, China) following the manufacturer’s instructions. Total RNA from GC cell lines and tissues was extracted using the TRIzol reagent (Invitrogen, Carlsbad, CA, United States), and reverse transcription was performed with the PrimeScript^TM^ RT reagent Kit with gDNA Eraser (Takara, Dalian, China) with random hexamers on 1 μg of RNA. Relative expression levels of FALEC and ECM1 were determined by qRT-PCR using the YBR^®^ Premix Ex Taq^TM^ II kit (Takara, Dalian, China) according to the manufacturer’s instructions. Primers used for qRT-PCR analyses were synthesized by GENEWIZ (Suzhou, China) and are listed in Supplementary Tables S2, S3. The relative expressions of interested genes were normalized to the expression of β-actin. Gene expression was analyzed using the 2^-ΔΔCt^ method, and all experiments were performed in triplicate.

### *In vitro* Cell Migration and Invasion Assays

Wound healing and transwell invasion and migration assays were performed as described previously (Cui et al., 2015). Briefly, a scratch was generated across confluent cell monolayers using a 10 μl sterile pipette tip followed by supplemented with serum-free medium after washed with phosphate buffered solution (PBS) to remove floating cells. *In vitro* cell migration and matrigel (BD Biosciences, San Jose, CA, United States) invasion assays were performed using the transwell system (8-μm pore size with polycarbonate membrane; Corning Costar, MA, United States). Cells were seeded into the top chamber in serum-free medium, and medium containing 10% fetal bovine serum was placed in the bottom chamber as an attractant. Cells migrating through the pores or invading through the matrigel were fixed with methanol and stained with 0.5% crystal violet after 36 h of incubation. Images were obtained at 20x magnification by using a microscope (Olympus, Tokyo, Japan).

### Cloning of pGL3-Promoter-*FALEC*-Reverse Reporters and Luciferase Assay

pGL3-Promoter was digested with BamHI and SalI, and FALEC coding sequence was synthesized by General Biosystems (General Biosystems, Inc., Chuzhou, China) was cloned into the restriction enzyme sites sites 5′ upstream to the luciferase gene. Luciferase assays were performed in 96-well plates using the Dual-Luciferase^®^ Reporter (DLR^TM^) Assay (Promega, Madison, WI, United States) according to the manufacturer’s protocol, and the relative luciferase activity was normalized to the Renilla luciferase activity.

### Western Blot Assay

Whole-cell lysates were separated by 10% sodium dodecyl sulfate–polyacrylamide gel electrophoresis and transferred to 0.22 μm polyvinylidene fluoride membranes (Millipore). After blocking the membrane with 5% skimmed milk for 2 h at room temperature, it was incubated with specific anti-ECM1 (Bioworld Technology, Inc.) and anti-β-actin (Sigma-Aldrich) antibodies overnight at 4°C. Protein detection was performed with the enhanced chemiluminescence (ECL) system (SuperSignal; Pierce, United States).

### Bioinformatic Analysis

Comparative analysis of *FALEC* and *ECM1* expression levels and correlation analysis were obtained from Gene Expression Profiling Interactive Analysis (GEPIA^1^, a web server for cancer and normal gene expression profiling and interactive analyses) (Tang et al., 2017). The expression of *ECM1* in GC and its correlation with clinical information such as GC grade and subtype was evaluated using online analysis tool UALCAN (Chandrashekar et al., 2017). Kaplan-Meier survival analysis^2^ was used to determine the influence of *ECM1* expression on GC prognosis, and the log-rank test was utilized to compare survival curves between high and low *ECM1* expression groups (Szasz et al., 2016).

### Statistical Analysis

All data are presented as means ± SD and all statistical analyses were performed using SPSS 20.0 software (IBM Corp., Armonk, NY, United States) or GraphPad Prism 5 software (GraphPad Software, Inc., La Jolla, CA, United States). Differences in the expression levels of *FALEC* and *ECM1* between tumor and non-tumor tissues were analyzed using the chi-squared test (χ2 test). The difference in *ECM1* expression regulated by *FALEC* was estimated using the Student’s *t*-test. The association between FALEC expression and pathological features of GC was analyzed with the chi-squared (χ2) test. The correlation between *FALEC* and *ECM1* expression levels was analyzed using Pearson’s correlation coefficient test. Survival curves were conducted using the Kaplan-Meier method and the difference was analyzed by log-rank test. All *p*-values presented were two-sided and the difference was considered to be statistically significant at ^∗^*p* < 0.05 and ^∗∗^*p* < 0.01.

## Results

### FALEC Was Upregulated in Human GC Cell Lines and Gastric Cancer Tissue Specimens

**FIGURE 1:**
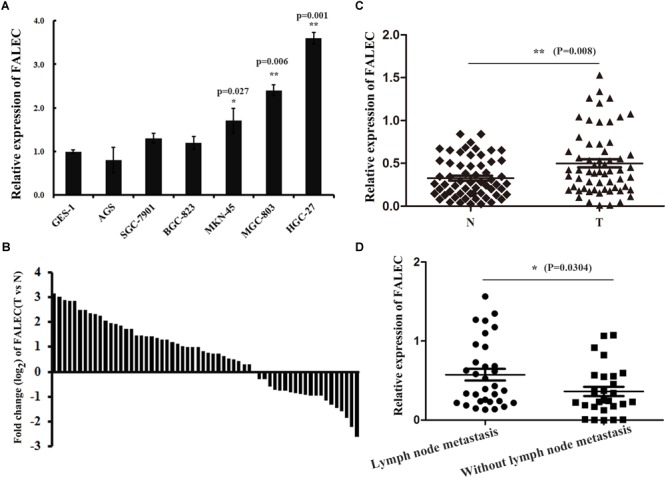
The expression of *FALEC* was upregulated in GC cell lines and tissues. **(A)** Relative expression of *FALEC* in GC cell lines and immortalized normal gastric epithelial cell line (GES-1) was examined by qRT-PCR. **(B)** The fold change (log2) of *FALEC* expression between 60 pairs of GC and adjacent normal tissues was measured by qRT-PCR. **(C)** *FALEC* expression in GC tissues and paired adjacent normal tissues were examined by qRT-PCR (*n* = 60), T (Tumor) and N (Non-tumor). **(D)** Patients with lymph node metastasis showed significantly higher levels of *FALEC* compared with no lymph node metastasis. ^∗^*P* < 0.05, ^∗∗^*P* < 0.01.

To investigate the possible role of *FALEC* in the progression of GC, we first detected the expression of *FALEC* in six human GC cell lines and 60 pairs of GC tissues and paired non-tumorous tissues. As shown by qRT-PCR analysis, *FALEC* expression was higher in GC cell lines and tumor tissues than controls and non-tumorous tissues (Figure 1A,B). The highest and lowest levels of FALEC were detected in HGC-27 (cell line established from lymph node with metastasis from GC) and AGS (established from human primary GC) cells indicated that FALEC may be a putative driver of metastasis (Figure 1A). Further statistical analysis found that *FALEC* expression was remarkably higher in tumor tissues compared with adjacent non-tumor tissues (Figure 1C). Next, we divided the samples into high (≥2-fold increase in GC tissues compared with adjacent non-tumor tissues) and low (less than 2-fold) *FALEC* expression groups to explore correlations between *FALEC* expression and clinicopathological characteristics in GC patients. As shown in Table 1, although no significant associations between *FALEC* expression and gender, age, Lauren’s classification, or histological type were detected, the expression of *FALEC* was significantly correlated with TNM and lymph node metastasis stage. An in-depth analysis found that *FALEC* expression significantly higher in GC tissues with lymph node metastasis than in non-lymph node metastasis (Figure 1D).

**Table 1:** The relationship of *FALEC* with the clinicopathological characteristics in patients with GC (*n* = 60).

Feature	Expression of *FALEC*		*F*	*p*-value
	T > N	T ≤ N		
Age			0.019	0.891
<60	12	9		
≥60	23	16		
Gender			0.099	0.753
Female	14	9		
Male	21	16		
Lauren’s classification			0.096	0.757
Intestinal type	14	11		
Diffuse type	21	14		
Lymph node metastasis			5.173	0.023*
No	12	16		
Yes	23	9		
Histological grade			5.813	0.055
High	6	10		
Moderate	9	8		
Poor	20	7		
TNM staging			6.251	0.012*
Stage I/II	11	16		
Stage III/IV	24	9		

*Chi-square test, ^∗^indicates *P*-value < 0.05.*

### Reduction of *FALEC* Expression Suppressed GC Cell Migration and Invasion *in vitro*

The amplification of a gene can lead to its overexpression such as EGFR and MYC are contained in frequently-amplified regions (Beroukhim et al., 2010). Hu et al. found that FALEC exhibited copy-number gains and losses in cancer (Athie and Huarte, 2014). But, we did not detect significant amplification of FALEC DNA (Supplementary Table S3), therefore, the overexpression of FALEC may not due to the DNA amplification in GC cells.

**FIGURE 2:**
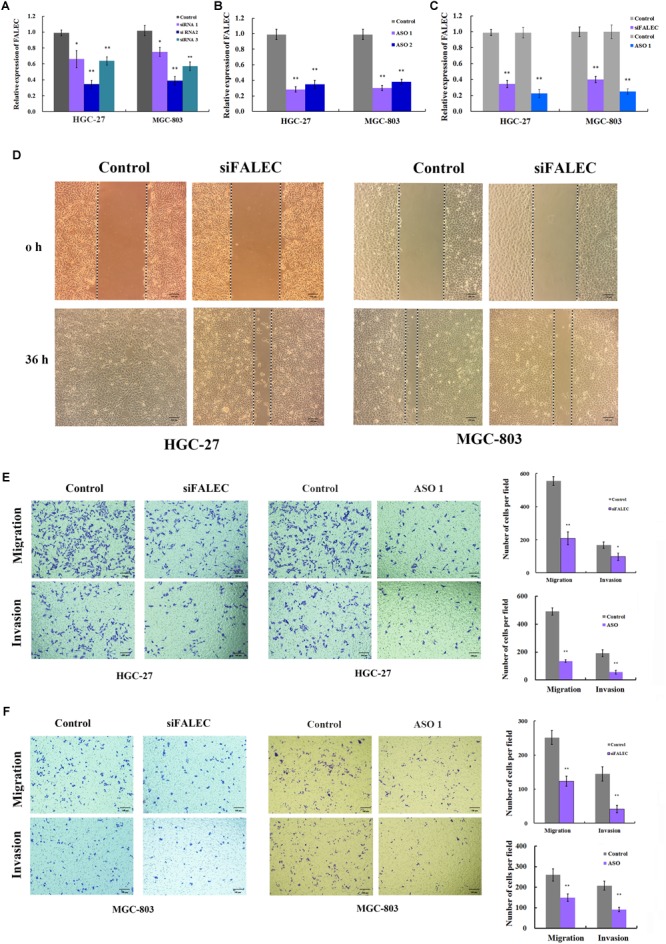
Knockdown of *FALEC* inhibited the cell migration and invasion in GC cells. **(A,B)** The interference efficiency of three siRNAs and two ASOs targeting to *FALEC* was analyzed by qRT-PCR in HGC-27 and MGC-803 cells. **(C)** The knockdown efficiency was measured by qRT-PCR when cell scratch and transwell assays were performed. **(D)** Knockdown of *FALEC* attenuated cell migration in HGC-27(left) and MGC-803 (right) cell lines. Images were acquired at 0 and 36 h after scratches were generated. Depletion of *FALEC* significantly inhibited HGC-27 **(E)** and MGC-803 **(F)** cells migration and invasion. Representative images of transwell results were taken under 100× original magnifications. The numbers of migrated or invaded cells were counted in five fields under the microscope from three independent experiments. ^∗^*P* < 0.05, ^∗∗^*P* < 0.01.

To explore the potential role of *FALEC* in GC progression, three siRNA pairs and two ASO targeting *FALEC* were synthesized to screen for the most effective sequence in HGC-27 and MGC-803 cells. The most efficient siRNA 2 and ASO 1 were chosen for subsequent functional analysis (Figure 2A,B). Because the over-expression of *FALEC* is significantly associated with lymph node metastasis in GC patients, we first explored its effect on the migration and invasion of GC cells. After transiently transfecting with siRNA or negative control into the GC cell lines, qRT-PCR was used to detect the interference efficiency before cell scratch and transwell assays were performed (Figure 2C). As shown in Figure 2D, the *FALEC* knockdown resulted in a slower recovery after wounding in HGC-27 (left) and MGC-803 (right) cells compared with the control. Also as expected, *FALEC* knockdown produced a significant reduction in migration and invasive ability in HGC-27 (Figure 2E) and MGC-803 (Figure 2F) cell lines compared with control by transwell assays. Taken together, these results indicate that knockdown of *FALEC* expression attenuates migration and invasion in GC cell lines, thereby suppressing the malignant progression of GC.

### Depletion of *FALEC* Attenuated Nearby *ECM1* Expression in GC Cell Lines

**FIGURE 3:**
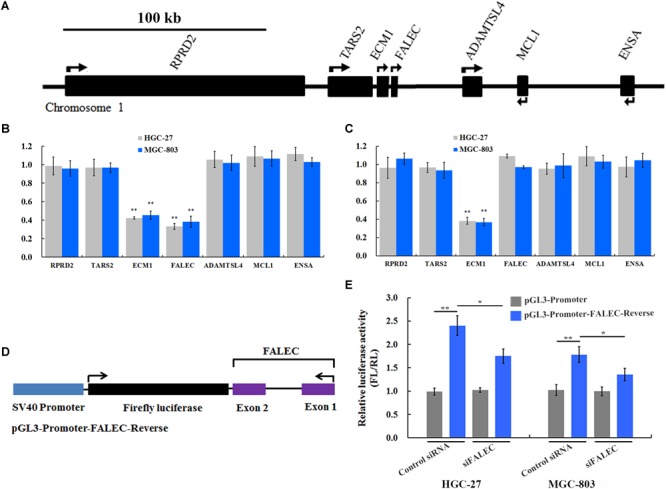
*FALEC* modulated *ECM1* expression by exerting its enhancer-like function in *cis.* **(A)** The relative loci of genes adjacent to *FALEC*. The scale bar represents 100 kb. **(B)** Depletion of *FALEC* specifically decreased expression of protein-coding gene *ECM1* in HGC-27 and MGC-803 cells. **(C)** siRNA-induced knockdown of the *ECM1* did not affect the transcription of its neighboring genes. ^∗∗^*P* < 0.01. **(D)** Diagram of the *FALEC* DNA sequence cloned down-stream of Firefly luciferase in a pGL3-Promoter vector (pGL3-Promoter-FALEC-Reverse). **(E)** *FALEC* inserts result in a significant enhancement of transcription in GC cells, and depletion of *FALEC* resulted in a significant decrease in transcriptional enhancement compared with control in HGC-27 and MGC-803 cells. ^∗^*P* < 0.05, ^∗∗^*P* < 0.01.

Previous studies demonstrated that a subset of enhancers was transcribed into a class of eRNAs, and that eRNA knockdown would decrease expression of nearby or target genes by *cis-*regulation (Kim et al., 2010; Li et al., 2013; Hsieh et al., 2014). To test this hypothesis, we interrogated the *FALEC* locus and found that protein-coding genes *TARS2, RPRD2, ECM1, ADAMTSL4, MCL1* and *ENSA* were within this genomic region (Figure 3A). To determine whether *FALEC* has an enhancer-like function, we explored the effect of *FALEC* on the transcription of nearby genes in HGC-27 and MGC-803 cell lines. As shown in Figure 3B, the depletion of FALEC led to a significantly reduced the expression of its adjacent protein-coding gene *ECM1*, but did not affect the other genes surrounding *FALEC* in mRNA level in HGC-27 and MGC-803 cells. These results implied that *FALEC* may functions as an enhancer element in specifically regulating the expression of *ECM1* by *cis*, and this effect was specific to the *ECM1*, as we did not detect any changes in the other genes surrounding FALEC. Moreover, *ECM1* knockdown did not affect the expression level of *FALEC* or any of the other adjacent genes further supporting the fact that *FALEC* is an independent regulator of *ECM1* (Figure 3C). Together, these results implied that *FALEC* might functions as an eRNA to activate *ECM1* expression in GC cell lines.

### *FALEC* Modulated the Expression of *ECM1* by Exerting Its Enhancer-Like Function

Previous studies showed that an important property of enhancers that stimulates transcriptional activity is independent of the orientation of the DNA sequence (Khoury and Gruss, 1983). To dissect the regulatory mechanisms of the *FALEC*-activation on the expression of *ECM1*, we constructed a vector in which the *FALEC* sequence is reversed (pGL3-Promoter-*FALEC*-Reverse) (Figure 3D) and cloned down-stream of Firefly luciferase in the pGL3-Promoter vector to assess its orientation independence and enhancement of transcription. As shown in Figure 3E, *FALEC* inserts result in a significant enhancement of transcription in HGC-27 and MGC-803 cells compared with control. To further demonstrate that the observed potentiation of gene expression is mediated through the action of *FALEC*, we knocked down the *FALEC* using siRNAs, depletion of *FALEC* resulted in a significant decrease of in transcriptional enhancement compared with control in HGC-27 and MGC-803 cells (Figure 3E). Taken together, these experiments demonstrated that *FALEC* modulated *ECM1* expression by exerting its enhancer-like function although the underlying nature of *FALEC* in the regulation of *ECM1* needs further investigation.

### Knockdown of *FALEC* Inhibited GC Cell Migration and Invasion Partly by Downregulating *ECM1*

**FIGURE 4:**
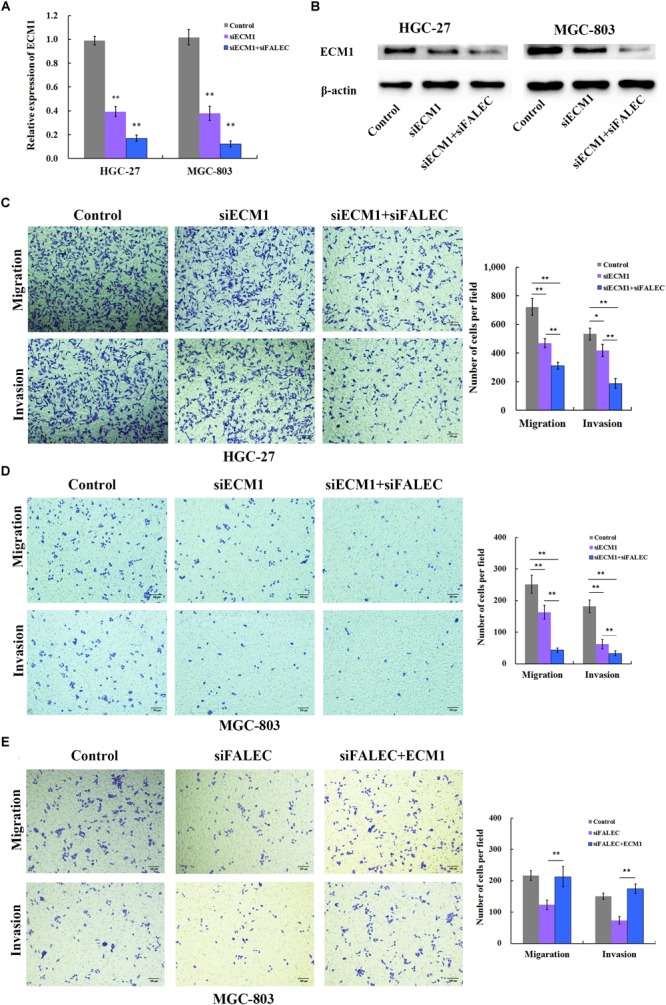
*FALEC* knockdown decreased migration and invasion partly by down regulating *ECM1* in GC cells. **(A,B)** Relative expression of *ECM1* was examined after *ECM1* silencing alone or simultaneously silencing *FALEC* and *ECM1* in HGC-27 and MGC-803 cells by qRT-PCR and western blotting assay. Compared with control, *ECM1* knockdown significantly impaired cell migration and invasion ability of HGC-27 and MGC-803 cells **(C,D)**. Similarly, the knockdown of *FALEC* and *ECM1* simultaneously resulted in significant decreased the migration and invasion in GC cells than sciencing *ECM1* alone or control group **(C,D)**. **(E)** *ECM1* overexpression markedly restored the abrogated migration and invasion of GC cells inhabited by *FALEC*.

Previous research suggested that *ECM1* plays pivotal roles in cancer cell migration and invasion (Xiong et al., 2012; Gomez-Contreras et al., 2017), so we examined whether *ECM1* affected the migration and invasion ability of the GC cells. SiRNA was used to knockdown the expression of ECM1, and the silencing efficiency was assessed by qRT-PCR (Figure 4A) and western blotting (Figure 4B) in HGC-27 and MGC-803 cells. As shown in Figure 4C,D, ECM1 knockdown significantly impaired the migration and invasion ability of GC cells compared with control. To further investigate whether *FALEC* affected cell migration and invasion by down-regulating *ECM1*, we used siRNA and transwell assays to evaluate the cell migration and invasion abilities of the two genes. Compared with the control group, simultaneous silencing of *ECM1* and *FALEC* significantly inhibited the migration and invasion ability of GC cells more than *ECM1* alone or the control group in GC cells (Figure 4C,D). These results indicated that *FALEC* knockdown inhibits cell migration and invasion partly by down-regulating *ECM1* in GC cells.

To clarify that *ECM1* was the functional target of *FALEC*, a rescue experiment was conducted in GC cells. We ectopically expressed *ECM1* by transfecting of pcDNA3.1(+) containing the *ECM1*-coding sequence together with siRNA for *FALEC* into MGC-803 cells. The transwell assay clearly demonstrated that *ECM1* overexpression markedly restored the abrogated migration and invasion of GC cells inhabited by *FALEC* (Figure 4E). This result further confirmed that *FALEC*-targeted *ECM1* is involved in GC cell migration and invasion.

### Elevated *ECM1* Expression Is Significantly Positively Associated With Increased *FALEC* Expression and Predicted Poor Prognosis in GC

**FIGURE 5:**
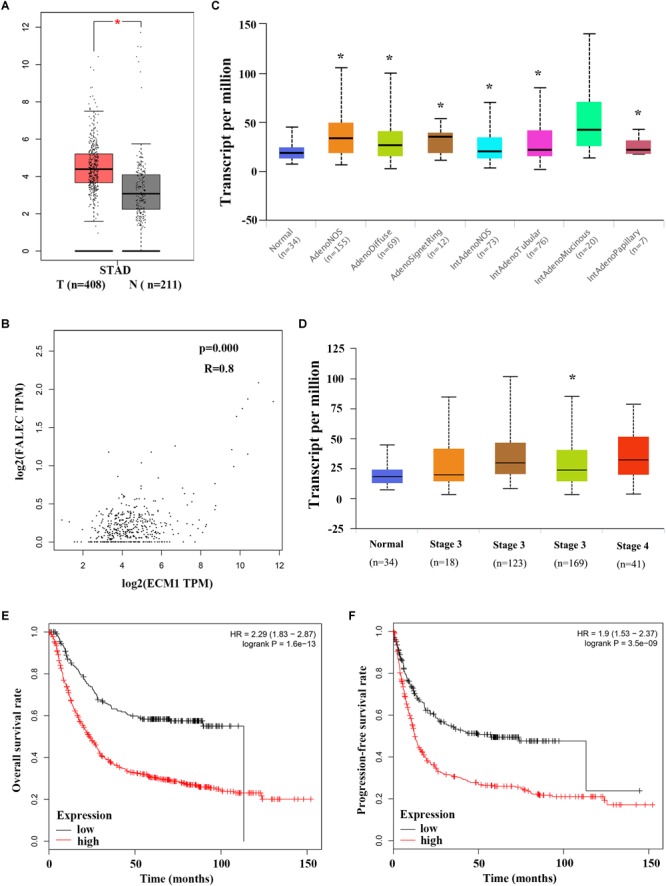
Elevated *ECM1* expressions is significantly positively associated with increased *FALEC* expression in patients with GC. **(A)** *ECM1* expression in GC tumor tissues was significantly higher than paired adjacent non-tumor samples, and a remarkable positive correlation was found between *ECM1* and *FALEC* mRNA expression in GC by using GEPIA database **(B)**. **(C,D)** shows a box plot that *ECM1* expression in different histological subtypes and stages in GC patients using UALCAN database. Kaplan-Meier survival plots shown that GC patients with high *ECM1* expression have reduced lower overall **(E)** and progression-free survival **(F)** rates compared with patients with low expression level of *FALEC*. ^∗^*P* < 0.05, ^∗∗^*P* < 0.01.

To explore the relationship between *ECM1* and FALEC mRNA expression and their clinical significance, we investigated *ECM1* expression levels in GC specimens using the GEPIA online database. As shown in Figure 5A, *ECM1* was significantly upregulated in tumor tissues compared with non-tumorous tissues. A remarkable positive correlation was found between *ECM1* and *FALEC* mRNA expression in GC (Figure 5B), further indicating that *FALEC* exerts enhancer effects to promote *ECM1* expression, thereby promoting migration and invasion in GC cells. Next, we employed the UALCAN online database to analyze the expression of *ECM1* in different stages and histological subtypes of GC patients. Box plot showing that the expression of *ECM1* in subtypes of GC samples were significantly higher than normal except for intestinal adenocarcinoma (Mucinous) (Figure 5C), and the expression level of *ECM1* was relatively high compared to the normal gastric tissues for GC patients (Figure 5D). Finally, we performed a correlation analysis of *ECM1* expression and clinical outcome of GC patients using a Kaplan–Meier plotter. Kaplan-Meier survival plots showed that high *ECM1* expression in GC patients was associated with shorter overall survival (OS) (Figure 5E) and progression-free survival rates (PFS) (Figure 5F) compared with patients with low *ECM1* expression. These results above suggested that FALEC exerts enhancer activity to promote the *ECM1* expression and participate in the development of GC.

## Discussion

Aberrant expression of lncRNAs that involved in the regulation of critical malignant biological behaviors such as cell proliferation and metastasis has been comprehensively reported in human cancers (Yan et al., 2015; Li et al., 2016; Hao et al., 2017; Chiu et al., 2018). However, although previous studies have demonstrated that abnormal lncRNA expression is involved in GC pathogenesis, but the regulators involved in lncRNAs dysregulation and underlying mechanisms in GC remained to be elucidated (Sun et al., 2016).

Long non-coding RNAs exert their regulatory functions in *cis* or in *trans* and are involved in various biological processes (Ulitsky and Bartel, 2013; Cech and Steitz, 2014). Previous studies revealed that *FALEC* contributed to carcinogenesis by *trans*-regulating the expression of genes such as *P21* and *AKT1* involved in the malignant proliferation, invasion, and metastasis of tumor cells (Hu X. et al., 2014; Zhong et al., 2015; Jeong et al., 2016; Ni et al., 2017; Pan et al., 2017; Zhao et al., 2017; Li B. et al., 2018; Li Y. et al., 2018; Wang et al., 2018). *FALEC* is a validated 566 nt lncRNA located at 1q21.2, which has been confirmed to be upregulated in carcinogenesis. However, little is known about the detailed function and mechanism of *FALEC* in *cis* in tumorigenesis and tumor development.

In our present study, we found the expression level of *FALEC* in GC tissues was significantly higher than in paired non-tumor tissues (Figure 1B). The high expression was positively correlated with lymph node metastasis and TNM stage (Table 1). Our results further proved that *FALEC* regulated the expression of *ECM1* by exerting its eRNA function in *cis*, thus affecting the migration and invasion ability of GC cells (Figure 2D, 3B,C,E, 4B–D).

Generally, eRNAs are transcribed from putative enhancer regions and play a critical role in the transcription of targeted genes (Heintzman et al., 2009), and the expression level of eRNAs is correlated with that of nearby genes (Mao et al., 2018). Our current research on *FALEC* and *ECM1* in GC supports these conclusions (Figure 5B). However, it remains to be determined how eRNAs regulate gene expression. Some evidence suggests that eRNAs affect chromatin states and enhancer–promoter looping, increase pol II binding, and act as a decoy for the negative elongation factor complex (Maruyama et al., 2014; Schaukowitch et al., 2014; Liang et al., 2016). These models remain controversial, and the underlying nature of eRNA in the regulation of gene expression requires further investigation. As for *FALEC*, our studies found that *FALEC* exerts its regulatory function in *cis* in GC, but the detailed mechanism of how *FALEC* regulates the expression of ECM1, and other targets may be influenced by *FALEC* in *cis* or in *trans* requires further investigation.

In summary, we showed that FALEC functions as an eRNA to activate *ECM1* expression in the progression of GC. *FALEC* could serve as a new potential biomarker and therapeutic target for individualized treatment of GC patients.

## Author Contributions

HW and ML conceived, designed the study, and wrote the manuscript. FQ, HW, and YZ performed the experiments and helped to draft the manuscript. JZ provided assistance for clinical sample collection, preservation, data and statistical analysis. SW, MH, TW, FH, WC, and DS provided statistical analysis of the data, contributed to some experiments, and helped to draft the manuscript.

## Conflict of Interest Statement

The authors declare that the research was conducted in the absence of any commercial or financial relationships that could be construed as a potential conflict of interest.

## Footnotes

1 http://gepia.cancer-pku.cn/

2 http://kmplot.com/analysis/index.php?p=service&cancer=gastric
