# Supplementary material for: Downregulation of Long Non-coding RNA FALEC Inhibits Gastric Cancer Cell Migration and Invasion Through Impairing ECM1 Expression by Exerting Its Enhancer-Like Function
Source: Front Genet. 2019 Mar 22;10:255. doi: 10.3389/fgene.2019.00255 (PMC6448009; doi:10.3389/fgene.2019.00255)
Supplement: Supplementary file 1 [file Data_Sheet_1.pdf]

Supplementary TABLE S1

TABLE S1 The list of the sequence of siRNAs and ASO

Gene	Sequence (5'→3')	
FALEC siRNA 1	sense	GUAGCAACUUCAAACGGAAUU
	antisense	UUCCGUUUGAAGUUGCUACUU
FALEC siRNA 2	sense	AGAACAUACAGGAGGAAGAUU
	antisense	UCUUCCUCCUGUAUGUUCUUU
FALEC siRNA 3	sense	GCGGAGACUUGUCUUUAAAUU
	antisense	UUUAAAGACAAGUCUCCGCUU
ECM1 siRNA	sense	CCGUCAGCAUGUGGUAUAUUU
	antisense	AUAUACCACAUGCUGACGGUU
siRNA-NC	sense	UUCUCCGAACGUGUCACGUUU
	antisense	ACGUGACACGUUCGGAGAAUU
FALEC ASO 1	target sequence	AAAAGGCCGGCCCAGCAATT
FALEC ASO 2	target sequence	CAAACGGAAGGTGTCAAAAG

Supplementary TABLE S2

TABLE S2 Primer sets were designed for qRT-PCR assays

Gene	Primer (5'→3')
β-actin	Forward: AAGACCTGTACGCCAACAC
	Reward: GTCATACTCCTGCTTGCTGAT
FALEC	Forward: CAAGCGGAGACTTGTCTTT
	Reward: GGCTGGTCTTGAACTCCTGA
ECM1	Forward: GCCCTCTGCTGTTACCTGAG
	Reward: CCTTGGCGTTCTCAGTGTCT
TARS2	Forward: GGATGCAGACTCTGGACTG
	Reward: CTTTGCTCTTTCTGGCCAAC
ADAMTSL4	Forward: CCCAGCCTGTGGATGTCTAT
	Reward: AAGGATTGGAGGAGGTGAA
MCL1	Forward: AAGGCGCTGGAGACCTTAC
	Reward: ATGTCCAGTTTCCGAAGCAT
ENSA	Forward: GGTCTTGGGTGTGATGTGTG
	Reward: GGCCTTTAGCTTTGCCTCTT

Supplementary TABLE S3

TABLE S3 Quantification of FALEC amplification by qRT-PCR

Cell lines	Fold amplification (FALEC/GAPDH)	*p* value
AGS	1.01	0.53
BGC-823	1.23	0.12
MKN-45	0.94	0.39
MGC-803	1.10	0.49
HGC-27	0.96	0.83

Note: Primer sequences for qRT-PCR were as follows: GAPDH forward: 5'-GCTGCTTTTAACTCTGGTAAAGTG-3', GAPDH reverse: 5'-TAGCACTCACCATGTAGTTGAG-3', generating a product of 79bp; FALEC forward: 5'-CTGTTTAACCCACCCTGTC -3', FALEC reverse: 5'- TTTTCTTCATCTCCCATTC-3', generating a product of 213 bp.
